# Taxifolin inhibits amyloid-β oligomer formation and fully restores vascular integrity and memory in cerebral amyloid angiopathy

**DOI:** 10.1186/s40478-017-0429-5

**Published:** 2017-04-04

**Authors:** Satoshi Saito, Yumi Yamamoto, Takakuni Maki, Yorito Hattori, Hideki Ito, Katsuhiko Mizuno, Mariko Harada-Shiba, Raj N. Kalaria, Masanori Fukushima, Ryosuke Takahashi, Masafumi Ihara

**Affiliations:** 1 Department of Regenerative Medicine and Tissue Engineering, National Cerebral and Cardiovascular Center, 5-7-1 Fujishiro-dai, Suita, Osaka 565-8565 Japan; 2 Department of Neurology, Kyoto University Graduate School of Medicine, Kyoto, 606-8507 Japan; 3 Department of Stroke and Cerebrovascular Diseases, National Cerebral and Cardiovascular Center, 5-7-1 Fujishiro-dai, Suita, Osaka 565-8565 Japan; 4 Department of CNS Research, Otsuka Pharmaceutical Co., Ltd, Tokushima, 771-0192 Japan; 5 Department of Drug Metabolism and Pharmacokinetics, Tokushima Research Institute, Otsuka Pharmaceutical Co., Ltd, Tokushima, 771-0192 Japan; 6 Department of Molecular Innovation in Lipidology, National Cerebral and Cardiovascular Center, Suita, 565-8565 Japan; 7 Institute for Ageing and Health, NIHR Biomedical Research Building, Newcastle University, Campus for Ageing and Vitality, Newcastle upon Tyne, NE4 5PL UK; 8 Department of MediScience, Translational Research Informatics Center, Foundation for Biomedical Research and Innovation, Kobe, 650-0047 Japan

**Keywords:** Alzheimer’s disease, Cerebral amyloid angiopathy, Oligomer, Taxifolin, Treatment

## Abstract

Cerebral amyloid angiopathy (CAA) induces various forms of cerebral infarcts and hemorrhages from vascular amyloid-β accumulation, resulting in acceleration of cognitive impairment, which is currently untreatable. Soluble amyloid-β protein likely impairs cerebrovascular integrity as well as cognitive function in early stage Alzheimer’s disease. Taxifolin, a flavonol with strong anti-oxidative and anti-glycation activities, has been reported to disassemble amyloid-β in vitro but the in vivo relevance remains unknown. Here, we investigated whether taxifolin has therapeutic potential in attenuating CAA, hypothesizing that inhibiting amyloid-β assembly may facilitate its clearance through several elimination pathways. Vehicle- or taxifolin-treated Tg-SwDI mice (commonly used to model CAA) were used in this investigation. Cognitive and cerebrovascular function, as well as the solubility and oligomerization of brain amyloid-β proteins, were investigated. Spatial reference memory was assessed by water maze test. Cerebral blood flow was measured with laser speckle flowmetry and cerebrovascular reactivity evaluated by monitoring cerebral blood flow changes in response to hypercapnia. Significantly reduced cerebrovascular pan-amyloid-β and amyloid-β_1-40_ accumulation was found in taxifolin-treated Tg-SwDI mice compared to vehicle-treated counterparts (*n* = 5). Spatial reference memory was severely impaired in vehicle-treated Tg-SwDI mice but normalized after taxifolin treatment, with scoring similar to wild type mice (*n* = 10–17). Furthermore, taxifolin completely restored decreased cerebral blood flow and cerebrovascular reactivity in Tg-SwDI mice (*n* = 4–6). An in vitro thioflavin-T assay showed taxifolin treatment resulted in efficient inhibition of amyloid-β_1-40_ assembly. In addition, a filter trap assay and ELISA showed Tg-SwDI mouse brain homogenates exhibited significantly reduced levels of amyloid-β oligomers in vivo after taxifolin treatment (*n* = 4–5), suggesting the effects of taxifolin on CAA are attributable to the inhibition of amyloid-β oligomer formation. In conclusion, taxifolin prevents amyloid-β oligomer assembly and fully sustains cognitive and cerebrovascular function in a CAA model mice. Taxifolin thus appears a promising therapeutic approach for CAA.

## Introduction

Cerebral amyloid angiopathy (CAA) is pathologically characterized by the deposition of amyloid-β within small cerebral vessels. CAA is a major cause of lobar intracerebral hemorrhage, cerebral infarction and cognitive impairment in the elderly, though there are currently no established treatments [18, 65, 69].

Amyloid-β deposition within cerebral capillaries has been consistently associated with the apolipoprotein E ε4 allele and is frequently concomitant with Alzheimer’s disease [66]. Accumulating lines of evidence have shown CAA plays a pivotal role in the pathogenesis of dementia [36, 47]. Structural network alterations and neurological dysfunction have been reported in CAA patients [10, 55]. CAA has been associated with Alzheimer’s disease in community-based older persons [8]. Recent pathological reports have shown that CAA is extremely common in sporadic Alzheimer’s disease [35], suggesting a strong, bidirectional relationship. Some forms of familial Alzheimer’s disease and Down syndrome also frequently exhibit CAA [56, 74]. CAA is commonly regarded as a pathological hallmark of Alzheimer’s disease, in parallel to senile plaque and neurofibrillary tangle deposition [17, 25].

Inhibiting amyloid-β assembly is a potential approach in the prevention of progression of both Alzheimer’s disease and CAA as soluble amyloid-β is likely to impair cerebrovascular integrity as well as cognitive function in the early stages of Alzheimer’s disease [21–23]. Cerebrovascular amyloidosis reduces the ability of vessels to constrict and dilate in response to physiologic stimulation, whereas impaired vasoreactivity predisposes ischemic damage [16, 20]. In addition to non-soluble forms, soluble amyloid-β deposition also induces cerebrovascular dysfunction, which is known to start before the appearance of visible amyloid-β deposits [22].

Silymarin, an extract of *Silybum marianum*, has been identified as a candidate in amyloid-β assembly inhibition. Silymarin has been reported to reduce amyloid-β plaque pathology in Alzheimer’s disease model mice [48]. However, no improvement in cognitive and cerebrovascular function was reported in subsequent experimental studies, potentially as silymarin is a mixture of several compounds, meaning its essential effector component remains obscure. Recently, taxifolin has been found to be the active component of silymarin, preventing amyloid-β aggregation in vitro [60]. Taxifolin, also known as a dihydroquercetin, is a catechol-type flavonoid with strong anti-oxidant and anti-glycation activities [19, 26]. We therefore hypothesized taxifolin would more effectively ameliorate cerebrovascular dysfunction due to CAA, rather than the senile plaques in Alzheimer’s disease, as only a small amount of its metabolites have been detected in the rat brain parenchyma after administration, suggesting low permeability across the blood-brain barrier (BBB) [76]. Here, we investigated whether taxifolin would modify the burden of CAA. Vascular amyloid-β accumulation is mainly composed of amyloid-β_1-40_, while senile plaques are characterized by amyloid-β_1-42_ accumulation. The pathogenicity of amyloid-β_1-40_ in CAA has been established in many previous experiments [49, 50, 53]. The purpose of this study was therefore to examine the potential protective effects of taxifolin on amyloid-β_1-40_ metabolism and cerebrovascular dysfunction in CAA model mice. This experimental approach may provide a rationale for subsequent clinical trials examining the efficacy of taxifolin in patients with CAA.

## Materials and methods

### Animals

We obtained heterozygous C57BL/6 J-Tg(Thy1-APPSwDutIowa)BWevn/J, also known as the Tg-SwDI, mice from Jackson Laboratory, Bar Harbor, USA. Low levels of the human *APP* gene with Swedish/Dutch/Iowa triple mutations are expressed in neurons under the control of the mouse *Thy1* promoter on a pure C57BL/6 J mice background [12]. Homozygous Tg-SwDI were generated from heterozygous Tg-SwDI mice and verified by backcrossing. Male homozygous Tg-SwDI and wild type (WT) mice aged 8 to 14 months (weighing 30–50 g) were examined in the present study. C57BL/6 J and C57BL/6 N mice were obtained from the Japan SLC, Hamamatsu, Japan. C57BL/6 J mice were used as WT controls, and C57BL/6 N mice aged 8 to 9 weeks were used for pharmacokinetic analysis only. Experimental mice were randomly assigned to taxifolin versus vehicle group and fed with pelleted chow containing 3% taxifolin (Ametis JSC, Blagoveshchensk, Russia) or standard pelleted chow only from the age of 1 month until death, unless stated otherwise. All mice were housed in a room with a 12-h light/dark cycle (lights on at 7:00 a.m.), with access to food and water *ad libitum*. No more than five mice were housed per cage, and were separated when fighting was noted. All study protocols were performed respecting animal dignity and were applicable to international and Japanese guidelines for the care, and the ethical standards of Kyoto University (Permit Number: MedKyo15274), and National Cerebral and Cardiovascular Center (Permit Number: 16068).

### Pharmacokinetic analysis

In the first cohort, male WT mice aged 8 weeks were given taxifolin solution once at 30, 100, 300 mg/kg of body weight by gavage into the stomach using a blunt-ended needle, followed by blood collection, via the vena cava, at 0.25, 0.5, 1.0, 2.0, 4.0, and 8.0 h after dosing. In the second cohort, mice aged 9 weeks were fed with pelleted chow containing either 1% or 3% taxifolin for 5 days. Blood was collected via the vena cava at 8:00, 13:00, 18:00, 23:00, and 3:00. Blood samples were collected with heparinized capillary tubes to prepare plasma, then allowed to clot for 30 min at room temperature before centrifugation for 10 min at 3000 × g to collect serum.

Levels of taxifolin in brain homogenates extracted from WT and Tg-SwDI mice were also investigated. The brains of Tg-SwDI mice aged 6 and 14 months were collected at 10:00 and 13:00, respectively. Tg-SwDI mice were fed with pelleted chow containing 3% taxifolin from the age of 1 month. WT and Tg-SwDI mice were deeply anesthetized by isoflurane inhalation and transcardially perfused with saline. Brains were harvested in saline and the homogenized lysates used for evaluation of taxifolin concentration. Taxifolin concentrations were measured using liquid chromatography/mass spectrometry/mass spectrometry. The limits of quantification in blood and brain were 3–10 ng/mL (9.9–33 nM) and 15–30 ng/g, respectively.

### Morris water maze test

Spatial reference memory was assessed using the Morris water maze test, as described earlier [27, 28]. A circular pool (diameter, 120 cm; depth, 40 cm) and a set of video analysis systems (EthoVision XT5; Noldus, Wageningen, Netherlands) were used. The pool was filled with water containing non-toxic white paint to a depth of 11 cm. A clear, circular platform (diameter, 10 cm) was submerged 1 cm below the water surface in the center of one quadrant of the pool (target quadrant). A red ‘cross’ sign and a blue ‘upward arrow’ (placed oppositely) were used as orientation cues to the swimming pool for the mice.

On the first 4 days, four trials per day were performed with a 30-minute interval between attempts (acquisition phase). The platform was kept in the same position during the acquisition phase. Mice were placed at the starting position (the quadrant adjacent to the target) and released into the water. Each mouse was allowed to swim for 60 s, discover the hidden platform, and climb onto it. The trial was immediately terminated after the mouse arrived on the platform or after 60 s had elapsed. If a mouse succeeded in climbing onto the platform, it was permitted to remain for 10 s. If a mouse did not reach the platform within 60 s, it was placed on the platform and allowed to remain for 15 s. Escape latency (time to goal) and total swimming distance to reach the platform were recorded.

On the fifth day, mice were subjected to a probe trial session where the platform was removed from the pool and mice allowed to swim for 60 s to search for it. The time spent in the platform quadrant and the number of entries into the target quadrant was recorded.

### Measurement of cerebral blood flow

Relative cerebral blood flow (CBF) of WT and Tg-SwDI mice was recorded using laser speckle flowmetry (Omegazone-2, Omegawave, Fuchu, Japan), as previously reported but with modifications [30, 42]. Laser speckle flowmetry obtains high-resolution, two-dimensional imaging and has a linear relationship with absolute CBF values [4]. Anesthesia was induced with 2%, and maintained with 1.5%, isoflurane in 80% nitrous oxide and 20% oxygen. An anesthesia mask for mice was used for isoflurane inhalation without tracheal intubation. The scalp was removed by a midline incision to expose the skull throughout CBF evaluation. CBF was measured in identically-sized regions of interest (circle 1 mm in diameter), located 1 mm posterior and 2 mm lateral from the bregma, corresponding to regions around Heubner’s anastomoses, connecting the dorsal branches of the anterior cerebral artery and the middle cerebral artery. Average CBF values in the bilateral hemispheres were recorded.

### Evaluation of vascular responses to hypercapnia

In order to examine cerebrovascular reactivity (CVR), the CBF response to hypercapnia was evaluated in WT and Tg-SwDI mice, with minor modifications to the methods described previously [29]. Mice were anesthetized with an intraperitoneal injection of α-chloralose (50 mg/kg) and urethane (750 mg/kg). The stability of anesthesia level was checked by testing corneal reflexes and motor responses to tail pinch. The trachea was intubated and mice mechanically ventilated at a stroke volume of 5 mL/kg body weight and ventilation rate of 100 strokes/minute with a ventilator. CBF was monitored by laser speckle flowmetry. To induce hypercapnia, mice were ventilated with 5% carbon dioxide for 5 min, followed by ventilation with 20% oxygen containing air. After measurement of baseline CBF, changes in response to hypercapnia were monitored for 5 min, with values obtained every 1 min.

### Histologic investigation

WT and Tg-SwDI mice were deeply anesthetized by an intraperitoneal injection of sodium pentobarbital (40 mg/kg) and transcardially perfused with 0.01 M phosphate buffered saline, followed by 4% paraformaldehyde in 0.1 M phosphate buffer. The removed brains were post-fixed in 4% paraformaldehyde overnight and embedded in paraffin, then sliced into 6 μm-thick sagittal sections 1 mm lateral from the midline. For thioflavin-S staining, sections were deparaffinized and immersed in a 100 μM thioflavin-S solution containing 50% ethanol for 30 min, then washed in 100% ethanol for 1 min. The fluorescent images were captured with a digital camera (BZ-9000, Keyence, Osaka, Japan). For Perls-Stieda’s iron staining, sections were immersed in a solution of an equal amount of hydrochloric acid and potassium ferrocyanide for 30 min, followed by counterstaining with 0.1% nuclear fast red for 3 min.

For immunohistochemistry, mouse anti-amyloid-β_1-16_ antibody (diluted 1:500, 6E10; BioLegend, San Diego, USA), rabbit anti-amyloid-β_1–40_ (diluted 1:500, FCA3340, Merck Millipore, Darmstadt, Germany), rabbit anti-amyloid-β_1–42_ (diluted 1:500, FCA3542, Merck Millipore) and rabbit anti-RAGE (receptor for advanced glycation end-products, receptor for AGEs) (diluted 1:100, ab3611, Abcam, Cambridge, UK) antibodies were used as primary antibodies after formic acid or heat-mediated antigen retrieval. The sections were subsequently treated with labeled polymer, prepared by combining amino acid polymers with peroxidase and secondary antibody, which is reduced to Fab’ fragment (Nichirei Biosciences, Tokyo, Japan). Sections were rinsed with phosphate buffered saline for 15 min between each step and finally visualized with 0.01% diaminobenzidine tetrahydrochloride and 0.005% H_2_O_2_ in 50 mM Tris-HCl (pH 7.6).

Densitometric analysis of amyloid-β accumulation was performed blindly to animal groups by setting regions of interest in the whole hippocampus in the immunostained sections. The percentage area of amyloid-β positive regions with the identical threshold was calculated using the Image-J software package (National Institutes of Health, Bethesda, USA).

The degree of RAGE expression in leptomeningeal and cortical arteries was classified into four grades: “none” (no expression), “low” (focal expression in less than 50% of whole circumference of vascular wall), “moderate” (intermediate expression in the vascular wall), and “high” (robust expression surrounding in more than 80% of the entire vascular wall). RAGE expression score was calculated from the average of qualified grading of RAGE expression (0 = none, 1 = low, 2 = moderate, 3 = high). Fifty leptomeningeal or cortical arteries (five per mouse) were analyzed in randomly selected regions in five vehicle-treated and five taxifolin-treated Tg-SwDI mice.

### Immunofluorescence for the evaluation of vascular amyloid-β accumulation

Tg-SwDI mice were deeply anesthetized with an intraperitoneal injection of sodium pentobarbital (40 mg/kg), then perfused transcardially with 0.01 M phosphate buffered saline, followed by 60 mg of fluorescein isothiocyanate (FITC) dextran in 0.9% saline, then 4% paraformaldehyde in 0.1 M phosphate buffer. Brains were removed and post-fixed in 4% paraformaldehyde for 12 h, then snap-frozen and sliced into 20 μm-thick sagittal sections at 1 mm lateral from the midline. Sections were then incubated at 4 °C overnight with a mouse anti-amyloid-β_1-16_ primary antibody (diluted 1:500), followed by fluorescent dye conjugated secondary antibody at room temperature for 60 min (diluted 1:200, Thermo Fisher Scientific, Waltham, USA).

### Thioflavin-T fluorescence assay

A SensoLyte Thioflavin T β-Amyloid (1–40) Aggregation Kit was used for the in vitro amyloid-β aggregation assay (AnaSpec, Fremont, USA). Thioflavin-T fluorescence assay measures change in the intensity of fluorescence emitted when amyloid fibrils or oligomers are bound to thioflavin-T [32, 33, 41]. Samples including 50 μM amyloid-β_1-40_ and thioflavin-T were incubated at 37 °C. Fluorescence intensity of each sample was recorded every 10 min for 6 h using a Wallac 1420 Multilabel counter (Perkin Elmer, Waltham, USA) with 440/486 nm excitation/emission filters set. Each sample was triply prepared and the mean fluorescence expressed in relative fluorescence units.

### Transmission electron microscopy (TEM)

The amyloid-β_1-40_ aggregates produced in the thioflavin-T assay were examined by electron microscope [43]. After 6-hour incubation at 37 °C, the samples were centrifuged at 16,000 × g at 4 °C for 90 min. The resultant pellets were suspended in distilled water and applied to a carbon film with 400 copper grids. The samples were negatively stained with 2% uranyl acetate for 2 min. Formation of fibrils was examined by electron microscopy (JEM-1200EX, JEOL, Akishima, Japan).

### Filter trap assay

Tg-SwDI mice were deeply anesthetized with an intraperitoneal injection of sodium pentobarbital (40 mg/kg) and transcardially-perfused with 0.01 M phosphate buffered saline. Whole frozen cerebrum were homogenized and harvested in Tris buffered saline (TBS, pH 7.5) and protease inhibitors cocktail (Nacalai Tesque, Kyoto, Japan). Soluble, extracellular-enriched proteins were collected from homogenized lysates after centrifugation at 100,000 × g at 4 °C for 60 min. The precipitate was again added with TBS and protease inhibitors, followed by centrifugation at 100,000 × g at 4 °C for 60 min. The precipitate was added with 70% formic acid and centrifuged at 100,000 × g at 4 °C for 60 min. The resultant supernatant was neutralized with 1 M Tris buffer (pH 11.0), which was used as the TBS insoluble fraction.

Filter trap assay was conducted, as described previously [40, 75]. Briefly, the protein concentration of the samples in TBS soluble fraction was measured and an equal amount of protein was subjected to vacuum filtration through a 96-well dot blot apparatus (Bio-Rad Laboratories, Hercules, USA) containing 200 nm pore-sized mixed cellulose ester membranes. The resultant membranes were then blocked by TBS containing 4% skim milk and incubated with primary antibody at 4 °C overnight. Mouse anti-amyloid-β_1-16_ antibody (diluted 1:1000, 6E10) and rabbit anti-amyloid-β oligomer antibody (diluted 1:1000, A11; Thermo Fisher Scientific, Waltham, USA) was used. The signal was visualized using horseradish peroxidase-conjugated anti-mouse and anti-rabbit IgG secondary antibodies (diluted 1:2000, Jackson Immuno Research Laboratories, West Grove, USA) with enhanced chemiluminescence (Merck Millipore). Immunoblot membranes were developed using the LAS-4000 Imaging System (Fujifilm, Tokyo, Japan). Densitometric measurement was performed using Image-J. Duplicate samples from each mouse were prepared for each primary antibody and the mean value measured.

### Enzyme-linked immunosorbent assay (ELISA)

The concentration of amyloid-β_1–40_, amyloid-β_1–42_ and amyloid-β oligomers in the TBS soluble fraction extracted from whole cerebrum of Tg-SwDI mice was estimated by ELISA [39]. The levels of amyloid-β_1–40_ and amyloid-β_1–42_ were also examined in the TBS insoluble fraction. The concentration of amyloid-β_1–40_, amyloid-β_1–42_ and amyloid-β oligomer (82E1-specific) was measured, according to the manufacture’s protocol (Human/Rat βAmyloid (40) ELISA Kit Wako II, Wako, Osaka, Japan; Human/Rat βAmyloid (42) ELISA Kit Wako, High-Sensitive; Human Amyloidβ Oligomers Assay Kit, Immuno-Biological Laboratories, Fujioka, Japan). Blood levels of amyloid-β_1–40_ and amyloid-β_1–42_ were also examined. Blood samples were obtained from the vena cava under isoflurane anesthesia, which were collected with heparinized capillary tubes to prepare plasma. The samples were allowed to clot for 30 min at room temperature followed by centrifugation for 5 min at 6000 × g to collect serum.

### Statistical analysis

All values were expressed as mean ± SD unless stated otherwise. Statistical analysis was conducted using Student’s *t* test or ANOVA followed by post hoc Turkey or Games-Howell tests. Differences with *p* < 0.05 were considered statistically significant in all analyses. Statistical analysis was performed using SPSS Statistics version 23.0 (IBM, Armonk, USA).

## Results

### Pharmacokinetics and safety profile of taxifolin

**Fig. 1:**
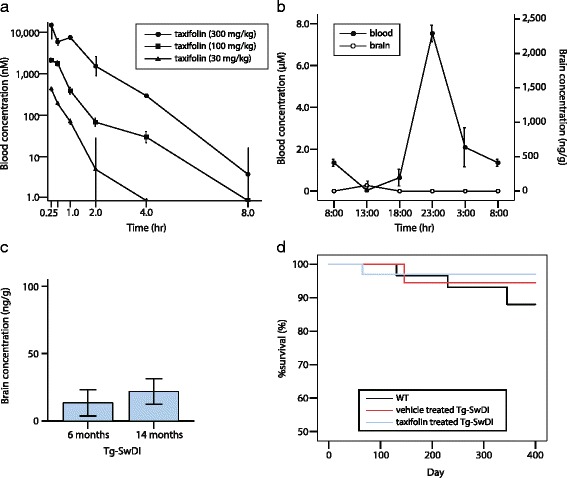
Pharmacokinetics and safety profile of taxifolin. Taxifolin is mostly degraded within an hour, and is rarely detected in the brain parenchyma. Survival rate is not affected by taxifolin treatment. **a** Line graphs showing changes in blood taxifolin concentration in 8-week-old WT mice receiving 30 mg/kg (*filled triangle*), 100 mg/kg (*filled square*), or 300 mg/kg (*filled circle*) of single taxifolin administration per oral (*n* = 3 for each). **b** Line graphs showing blood and brain taxifolin concentration in 9-week-old WT mice fed with the pelleted chow containing 3% taxifolin (*n* = 3). The first vertical axis represents blood concentration (*filled circle*). The second axis represents brain concentration (*open circle*). **c** A histogram showing brain taxifolin concentration in Tg-SwDI mice with taxifolin treatment at the age of 6 and 14 months (*n* = 5–6 for each groups). **d** Kaplan-Meier plots showing taxifolin does not affect survival rate of WT (*black line*) and Tg-SwDI mice treated with vehicle (*red line*) or taxifolin (*blue line*) (*n* = 28–33 for each group). Error bars indicate ± SE

We firstly investigated the pharmacokinetic profile of taxifolin in 8-week-old WT mice given by solution orally via gavage. Blood levels of taxifolin were increased in a dose-dependent manner (Fig. 1a). In 300 mg/kg taxifolin-treated mice, maximum concentration (Cmax) and elimination half-life period were recorded as 18.87 μM (5741 ng/mL) and 0.67 h, respectively, as in previous rat studies [70].

We evaluated changes in taxifolin concentrations in 9-week-old WT mice receiving each type of pelleted chow: 1% or 3% taxifolin-containing food. Although a previous investigation suggested more than 10 μM of taxifolin was needed to disassemble amyloid-β in vitro [61, 72], the Cmax of 1% taxifolin-treated mice was 0.199 μM (60.52 ng/mL), meaning 1% taxifolin would not be sufficient to achieve amyloid-β disaggregation in vivo. In contrast, blood taxifolin levels in 3% taxifolin-treated WT mice were substantially elevated and Cmax recorded as 7.53 μM (2290 ng/mL) (Fig. 1b). Blood taxifolin levels in Tg-SwDI mice receiving 3% taxifolin treatments were almost equivalent to those in 3% taxifolin-treated WT mice (data not shown); we therefore administered pelleted chow containing 3% taxifolin in subsequent experiments. We also estimated taxifolin levels in brain homogenates extracted from WT and Tg-SwDI mice treated with 3% taxifolin (Fig. 1b and c). The estimates suggested the dose of taxifolin was too low to reach a sufficient level in the brain parenchyma.

The safety profile of taxifolin has been studied previously [7, 79]. Consistent with these reports, animal survival rates were comparable among vehicle- or taxifolin-treated WT and Tg-SwDI mice groups (Fig. 1d). Taxifolin treatment did not induce any apparent systemic abnormalities in Tg-SwDI mice.

### Complete normalization of spatial reference memory impairment in taxifolin-treated Tg-SwDI mice

**Fig. 2:**
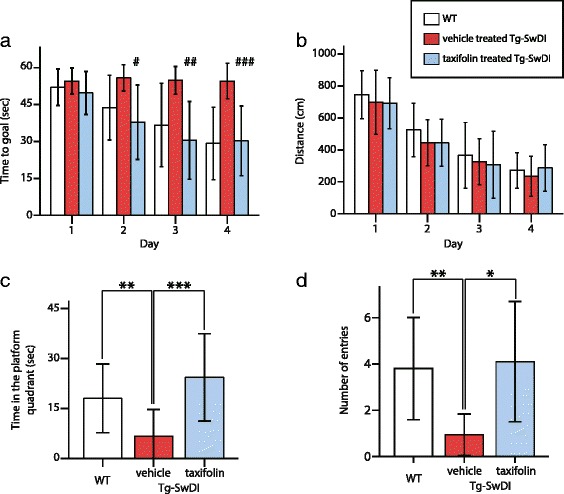
Effects of taxifolin treatment on cognitive function in the Morris water maze test. Spatial reference memory impairment in Tg-SwDI mice was completely normalized in taxifolin-treated Tg-SwDI mice. (**a** and **b**) Time course of escape latency (time to goal) (**a**) and total swimming distance (**b**) in acquisition phase recorded in vehicle-treated WT (*white*), vehicle-treated Tg-SwDI (*red*), and taxifolin-treated Tg-SwDI mice (*blue*) at 8-months-old receiving taxifolin or vehicle for 7 months (*n* = 10–17 for each group). (**c** and **d**) The time spent in the target quadrant (**c**) and the number of entries into target quadrant (**d**) in the probe trial. Error bars indicate ± SD. #*p* < 0.05 in WT versus vehicle-treated Tg-SwDI mice and in vehicle-treated versus taxifolin-treated Tg-SwDI mice. ##*p* < 0.01 in WT versus vehicle-treated Tg-SwDI mice and in vehicle-treated versus taxifolin-treated Tg-SwDI mice. ###*p* < 0.001 in WT versus vehicle-treated Tg-SwDI mice and *p* < 0.01 in vehicle-treated versus taxifolin-treated Tg-SwDI mice. *p < 0.05, **p < 0.01, ***p < 0.001

We examined whether taxifolin treatment affected spatial learning and reference memory impairment by performing the Morris water maze test in 8-month-old WT and Tg-SwDI mice. During the acquisition phase, compared with WT, vehicle-treated Tg-SwDI mice exhibited significantly longer escape latencies suggesting severely impaired reference memory. However, the latency in taxifolin-treated Tg-SwDI mice was comparable to WT mice (Fig. 2a). Recorded total swimming distance did not differ among WT, vehicle-treated Tg-SwDI, and taxifolin-treated Tg-SwDI, mice (Fig. 2b). In the probe trial phase, the time present in the platform quadrant and the number of entries into the target quadrant in WT and taxifolin-treated Tg-SwDI mice was comparable and both values were significantly greater than those in vehicle-treated Tg-SwDI mice (Fig. 2c and d), indicating taxifolin completely normalized spatial reference memory impairment in Tg-SwDI mice.

### Restoration of CBF and CVR in taxifolin-treated Tg-SwDI mice

**Fig. 3:**
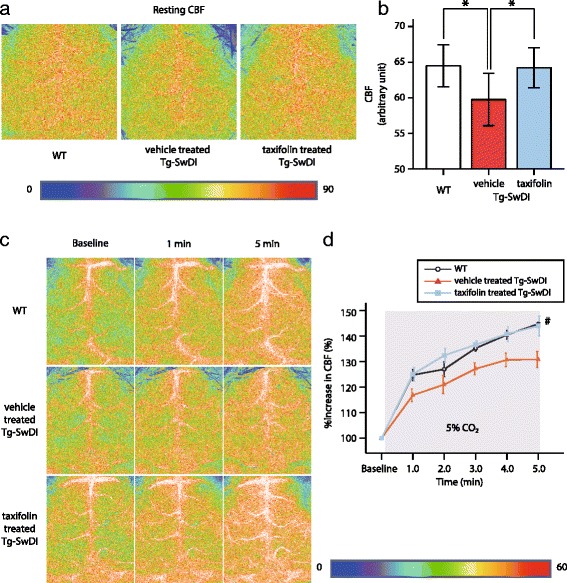
Effects of taxifolin treatment on CBF and CVR in Tg-SwDI mice. Taxifolin restored CBF reduction and impaired CVR caused by vascular amyloid-β in Tg-SwDI mice. **a** Representative images showing resting CBF measured by laser speckle flowmetry in vehicle-treated WT (*left*), vehicle-treated (*middle*), and taxifolin-treated Tg-SwDI (*right*), mice at 12 months of age receiving taxifolin or vehicle for 11 months. **b** A histogram showing CBF (*n* = 7–13 for each group). **c** Representative images of CBF levels monitored by laser speckle flowmetry before (left column; baseline) and 1 min (middle column) and 5 min (right column) after hypercapnia in vehicle-treated WT (*upper row*), vehicle-treated (*middle row*) and taxifolin-treated, Tg-SwDI mice (*lower row*) at 12 months of age receiving taxifolin or vehicle for 11 months. **d** Temporal profiles of %increase in CBF in WT (*black open circle*) and Tg-SwDI mice treated with vehicle (*red*-filled triangle) or taxifolin (*blue*-filled square) (*n* = 4–6 for each group). Error bars indicate ± SD (**b**) and ± SE (**d**). *p < 0.05 and #*p* < 0.05 in WT versus vehicle-treated Tg-SwDI mice and in vehicle-treated versus taxifolin-treated Tg-SwDI mice

Impairments in cerebral circulation and vascular reactivity have substantial roles in the onset and progression of cognitive dysfunction in patients with CAA and several animal models [16, 42]. We firstly investigated resting CBF of WT and Tg-SwDI mice at 12 months of age using laser speckle flowmetry. Vehicle-treated Tg-SwDI mice exhibited significantly decreased CBF compared with WT mice (Fig. 3a; left and middle panels). However, taxifolin treatment restored CBF reduction in Tg-SwDI mice (Fig. 3a; right panel). Resting CBF of taxifolin-treated Tg-SwDI mice was comparable to that of WT mice (Fig. 3b).

We then evaluated the cerebral hemodynamic response to hypercapnia, which is attributable to non-specific vasodilator action via the relaxation of vascular smooth muscle cells [78]. Baseline CBF was measured under tracheal intubation, immediately before inhalation of 5% carbon dioxide. Because of the difference of anesthetic drugs, baseline CBF (Fig. 3c; left panels) was lower than resting CBF (Fig. 3a). Once inhalation of 5% carbon dioxide was started, CBF rapidly increased in all mice. However, the degree of CBF response to hypercapnia was significantly decreased in vehicle-treated Tg-SwDI mice compared with WT mice (Fig. 3c; upper and middle rows). The CBF response to hypercapnia in taxifolin-treated Tg-SwDI mice was nearly equivalent to that in WT mice (Fig. 3c; lower row and 3d), suggesting taxifolin abolished the impaired CVR in Tg-SwDI mice.

### No effects on CBF and CVR by taxifolin treatment in WT mice

**Fig. 4:**
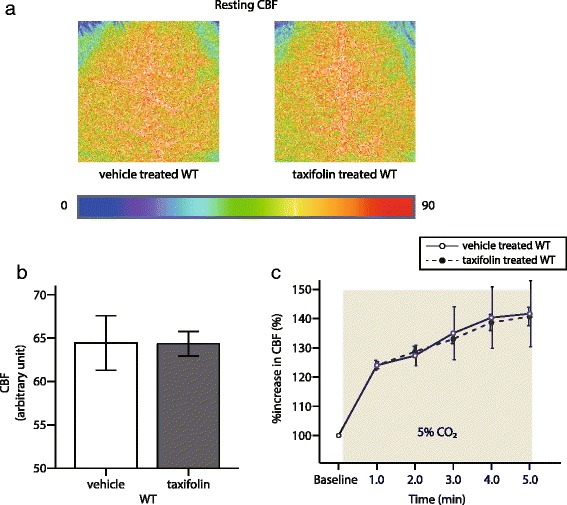
Effects of taxifolin treatment on CBF and CVR in WT mice. CBF and CVR was not changed between vehicle and taxifolin treated WT mice. **a** Representative images showing resting CBF measured by laser speckle flowmetry. In vehicle-treated (*left*), and taxifolin-treated (*right*), WT mice at 8 months of age receiving vehicle or taxifolin for 7 months. **b** A histogram showing CBF (*n* = 5–8 for each). **c** Temporal profiles of %increase in CBF in WT mice aged 8 months with vehicle (*open circle*) or taxifolin (*filled circle*) for 7 months (*n* = 3 each). Error bars indicate ± SD (**b**) and ± SE (**c**)

Taxifolin and other flavonols were reported to possess several protective effects on cerebrovasculature [62, 68]. Taxifolin ameliorated cerebral infarction induced by middle cerebral arterial occlusion through reducing oxidative stress in rats [71]. In order to examine the additive effects of taxifolin on vessels without amyloid-β deposits, resting CBF and CBF response to hypercapnia (namely, CVR) was evaluated in taxifolin-treated and vehicle-treated WT mice at 8 months of age. Both mice groups showed comparable levels of resting CBF (Fig. 4a and b) and CVR (Fig. 4c), suggesting that taxifolin did not exert positive effects on intact vessels.

### Decreased vascular amyloid-β and amyloid-β_1-40_ deposits in taxifolin-treated Tg-SwDI mice

**Fig. 5:**
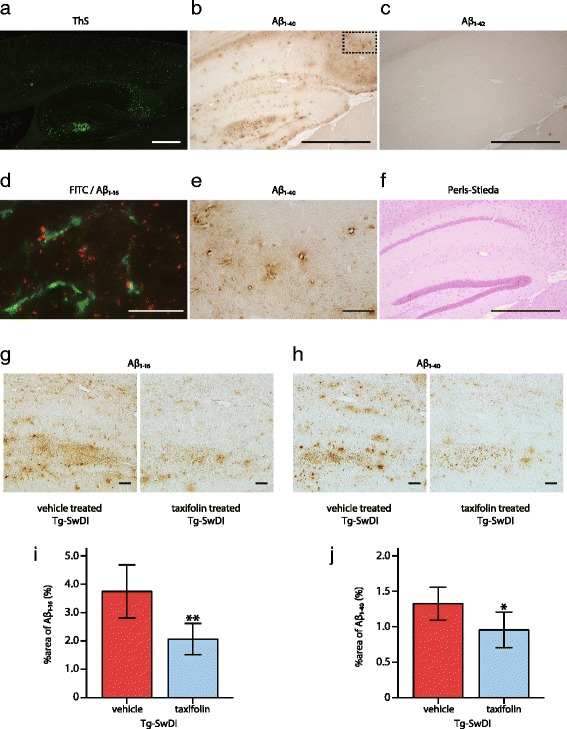
Effects of taxifolin treatment on amyloid-β pathology in Tg-SwDI mice. Vehicle-treated homozygote Tg-SwDI mice exhibited cerebrovascular amyloid-β accumulation, which was most abundantly observed in the hippocampus with predominance of amyloid-β_1-40_, rather than amyloid-β_1-42_, without apparent microhemorrhages or infarcts. Amyloid-β deposits were decreased in taxifolin-treated Tg-SwDI mice compared to vehicle-treated Tg-SwDI mice. **a** Representative image of thioflavin-S staining. Lower magnification of the cortex and the hippocampus in the 12-month-old vehicle-treated Tg-SwDI mice. (**b**, **c**, **e**) Representative images of amyloid-β_1-40_ and amyloid-β_1-42_ immunostaining in the hippocampus in 8-month-old vehicle-treated Tg-SwDI mice. **b** Lower magnification image of amyloid-β_1-40_ immunostaining. **c** Amyloid-β_1-42_ immunostaining. **e** Higher magnification of amyloid-β_1-40_ accumulating vessels (corresponding to dashed square in panel **b**). **d** Representative microscopic fluorescent images of amyloid-β_1-16_ immunostaining (*red*) in the hippocampus of the 12-month-old Tg-SwDI mice after FITC dextran (*green*) injection. Almost all of the amyloid-β_1-16_ accumulation is adjacent to cerebral small vessels. **f** Representative images of Perls-Stieda’s iron staining in the 8-month-old vehicle-treated Tg-SwDI mice. (**g** and **h**) Representative images of amyloid-β_1-16_ (**g**) and amyloid-β_1-40_ (**h**) in the hippocampus of 8-month-old Tg-SwDI mice treated with vehicle (*left*) or taxifolin (right) for 7 months. (**i** and **j**) Histograms showing the density of regions immunoreactive for amyloid-β_1-16_ (**i**) and amyloid-β_1-40_ (**j**) in the hippocampus of 8-month-old Tg-SwDI mice treated with vehicle or taxifolin (*n* = 5 each). Scale bars indicate 500 μm (**a**, **b**, **c**, **f**), and 50 μm (**d**, **e**, **g**, **h**). Error bars indicate ± SD. *p < 0.05 and **p < 0.01

Amyloid-β deposits in Tg-SwDI mice were most abundantly observed in the hippocampus (Fig. 5a) and characterized by predominant amyloid-β_1-40_ (Fig. 5b), rather than amyloid-β_1-42_ accumulation (Fig. 5c). The deposits were distributed in the perivascular areas of cerebral small arteries (Fig. 5d and e), which replicated the distribution of amyloid-β deposits observed in CAA patients [66].

Immunohistochemical analysis was performed in order to determine whether taxifolin ameliorated cerebrovascular amyloid-β accumulation in vivo. Compared with vehicle-treated Tg-SwDI mice, taxifolin-treated Tg-SwDI mice at 8 months of age showed significantly reduced immunoreactivity of both amyloid-β_1-16_ (Fig. 5g and i) and amyloid-β_1-40_ (Fig. 5h and j).

Because cerebral microinfarcts and hemorrhages are commonly observed in CAA patients, we performed H&E and Perls-Stieda’s iron staining as well as immunostaining for glial fibrillary acidic protein and ionized calcium binding adaptor molecule 1: no ischemic or hemorrhagic lesions were observed in taxifolin-treated and vehicle-treated Tg-SwDI mice until 12 months of age (Fig. 5f), in accordance with our previous report [51].

### No effect on cerebrovascular RAGE expression by taxifolin treatment

**Fig. 6:**
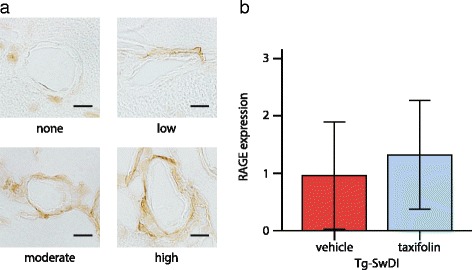
Effects of taxifolin treatment on cerebrovascular RAGE expression in Tg-SwDI mice. Taxifolin treatment did not significantly change the level of cerebrovascular RAGE expression in Tg-SwDI mice. **a** Representative images of RAGE immunostaining. **b** Histogram showing the grade of cerebrovascular RAGE expression in 8-month-old Tg-SwDI mice receiving vehicle or taxifolin for 7 months. Twenty five leptomeningeal and cortical arteries (five per mouse) for each group. Scale bars indicate 10 μm. Error bars indicate ± SD

Prior work has demonstrated *Silybum marianum* suppresses formation of advanced glycation end products (AGEs) [64], and marked anti-glycation activity is a unique property of taxifolin [26]. As RAGE is the major amyloid-β influx receptor in the BBB, transporting amyloid-β from the blood into the brain, RAGE inhibition was predicted to ameliorate Alzheimer’s disease and CAA [14, 15]. We assessed whether taxifolin reduces amyloid-β pathology by decreasing RAGE expression in Tg-SwDI mice at 8 months. Cerebrovascular RAGE immunoreactivity was found to be comparable between vehicle- and taxifolin-treated groups (Fig. 6a and b).

### Amyloid-β_1-40_ disassembly by taxifolin treatment

**Fig. 7:**
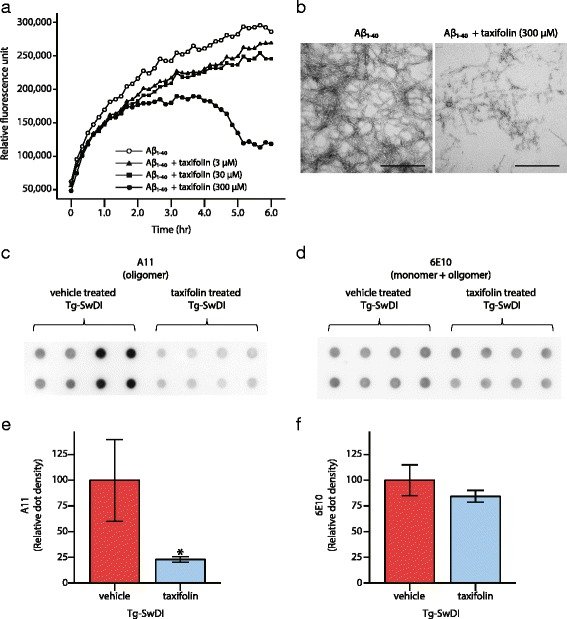
Effects of taxifolin treatment on amyloid-β assembly. Efficient inhibition of amyloid-β_1–40_ assembly by taxifolin was shown both in vitro and in vivo. **a** Amyloid-β_1–40_ gradually formed aggregation bound with thioflavin-T in the absence of taxifolin (*open circle*). However, taxifolin treatment inhibited amyloid-β_1-40_ assembly (addition of 3 μM of taxifolin: filled triangle, 30 μM of taxifolin: filled square, 300 μM of taxifolin: *filled circle*). The mean fluorescence of triply prepared samples was expressed. **b** TEM images of fibrils after 6-hour incubation of amyloid-β_1-40_ alone (*left*) and amyloid-β_1-40_ with 300 μM of taxifolin (*right*). **c**–**f** The results of filter trap assay show taxifolin-treated Tg-SwDI mice have lower levels of amyloid-β oligomer than vehicle-treated Tg-SwDI mice. (**c** and **d**) The blot images of amyloid-β in the TBS soluble fractions extracted from the brains of 9-month-old Tg-SwDI mice receiving vehicle or taxifolin for 8 months (*n* = 4 each). The amyloid-β oligomers (**c**) and total amyloid-β (**d**) was detected by anti-amyloid-β oligomer antibody (A11) and anti-amyloid-β_1-16_ antibody (6E10). The duplicate samples were prepared independently. (**e** and **f**) Densitometric analysis of amyloid-β oligomer (**e**) and total amyloid-β (**f**). Scale bars indicate 500 nm. Error bars indicate ± SD. *p < 0.05

Preferential accumulation of amyloid-β_1-40_ over amyloid-β_1-42_ is commonly observed in CAA and in Tg-SwDI mice. Therefore, we assessed the effects of taxifolin on amyloid-β_1-40_ assembly with an in vitro thioflavin-T fluorescence assay. In the absence of taxifolin, amyloid-β_1–40_ gradually formed aggregates that bound with thioflavin-T (Fig. 7a). However, the addition of taxifolin decreased thioflavin-T fluorescence intensity, suggesting efficient amyloid-β_1-40_ disassembly by taxifolin in vitro. Notably, the addition of 300 μM taxifolin resulted in marked amyloid-β_1-40_ disassembly. The inhibition of amyloid-β_1-40_ fibril formation by taxifolin was also confirmed by TEM imaging (Fig. 7b).

In order to confirm the amyloid-β disassembly by taxifolin in vivo, we conducted a filter trap assay and estimated the concentrations of total amyloid-β and amyloid-β oligomers in TBS soluble fractions of 9-month-old Tg-SwDI mice. Anti-oligomer antibody (A11) binds to the amyloid-β oligomer only, while the anti-amyloid-β_1-16_ antibody (6E10) detects both the amyloid-β monomer and oligomer. The levels of amyloid-β oligomers were significantly reduced in taxifolin-treated, compared with vehicle-treated, Tg-SwDI mice (Fig. 7c and e), whereas total soluble amyloid-β load was comparable between the two groups (Fig. 7d and f).

### Amyloid-β oligomer disassembly and promotion of amyloid-β clearance into blood

**Fig. 8:**
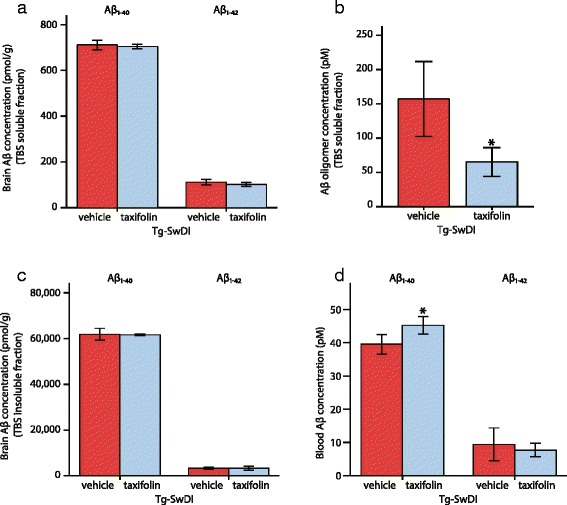
Effects of taxifolin treatment on oligomer formation and amyloid-β clearance. Tg-SwDI mice aged 14 months, which received taxifolin for 13 months, exhibited significant decrease in amyloid-β oligomers in the brain and increase of amyloid-β_1-40_ clearance into the circulation compared with vehicle-treated Tg-SwDI mice, while the levels of amyloid-β_1-40_ and amyloid-β_1-42_ in the TBS soluble and insoluble fractions were comparable between vehicle- and taxifolin-treated groups. **a**–**d** The amount of amyloid-β_1-40_, amyloid-β_1-42_, and amyloid-β oligomers analyzed by ELISA. **a** The amount of amyloid-β_1-40_ and amyloid-β_1-42_ in the TBS soluble fraction. **b** Amyloid-β oligomers in the soluble fraction. **c** Amyloid-β_1-40_ and amyloid-β_1-42_ in the insoluble fraction (a-c: *n* = 4–5 each). **d** The levels of amyloid-β_1-40_ and amyloid-β_1-42_ in the blood (amyloid-β_1-40_: *n* = 4–6 each, amyloid-β_1-42_: *n* = 3 each). Error bars indicate ± SD. *p < 0.05

We performed ELISA for amyloid-β_1-40_, amyloid-β_1-42,_ and amyloid-β oligomers measurement in TBS soluble and insoluble fraction extracted from 14-month-old Tg-SwDI mice receiving vehicle or taxifolin. No significant difference was recorded in the concentration of amyloid-β_1-40_ and amyloid-β_1-42_ in TBS soluble fraction between vehicle- and taxifolin-treated groups (Fig. 8a); however, the level of amyloid-β oligomers was significantly reduced in taxifolin-treated Tg-SwDI mice, suggesting that taxifolin prevented amyloid-β oligomer formation (Fig. 8b). Neither amyloid-β_1-40_ nor amyloid-β_1-42_ concentration in TBS insoluble fraction was changed by taxifolin treatment (Fig. 8c). The serum level of amyloid-β_1-40_ level, but not of amyloid-β_1-42_, was significantly elevated in 14-month-old Tg-SwDI mice receiving taxifolin compared to vehicle treated Tg-SwDI mice (Fig. 8d). These results indicate that taxifolin suppressed amyloid-β assembly by inhibiting oligomer formation, which increased amyloid-β clearance, preferably amyloid-β_1-40_, into the systemic circulation.

## Discussion

Our study revealed taxifolin inhibited amyloid-β_1–40_ assembly and oligomer formation, leading to facilitated clearance of amyloid-β into the circulation, restoration of CBF and CVR, reduction of amyloid-β accumulation and normalization of spatial reference memory impairment in Tg-SwDI, a widely-used CAA mouse model. To our knowledge, this is the first report showing a reduction in the amyloid-β oligomer ameliorates the phenotypic expression of CAA, suggesting taxifolin holds potential as a novel therapeutic intervention for CAA.

Taxifolin represents a minor fraction of the flavonols contained in silymarin, typically comprising of approximately 50% silybin, 20% silychristin, 10% silydianin, 5% isosilybin and an unidentified organic polymer fraction formed from the above compounds [2]. Ability to inhibit amyloid-β_1–42_ assembly and to dissolve amyloid-β_1–42_ fibrils was reported in taxifolin, but such effects were not observed with silybin, silychristin, and silydianin [60]. Taxifolin is also known to possess multiple pharmacological actions such as anti-oxidation [19], AGE formation suppression [26], and mitochondrial protection [24], and has received increasing attention because of its potential in the treatment of various diseases including malignancy, cardiovascular diseases, chronic hepatitis, hyperlipidemia and neurocognitive disorders [72]. The pharmacokinetic profile of taxifolin has provided essential data, which can be applied to future investigations of various diseases, where such information is lacking.

We detected only small amounts of taxifolin in the brains of both WT and Tg-SwDI mice receiving drug treatment. In order to inhibit both amyloid-β production in neural cells and parenchymal amyloid-β plaques, drugs need to pass through the BBB, composed of capillary endothelial cells, surrounded by pericytes, basal lamina and the end feet of astrocytic processes [1]. However, drugs aiming for maintenance of cerebrovascular integrity, including taxifolin, need not reach neurons through the BBB [42], providing advantage for clinical application as drug delivery is a frequent problem in the development of novel therapies for neurological diseases.

We administered taxifolin in pelleted chow because of its short elimination half-life. However, the estimated levels of taxifolin were not consistent, and much lower in the daytime than night, suggesting a consistent diurnal concentration of taxifolin is not required to achieve amyloid-β disassembly. The low taxifolin concentration in the daytime is likely to result from lower daytime activity and food intake [38]. We did not monitor the timing of food intake, but an earlier report has shown food intake of mice peaks within 6 h after turning off the light, which was consistent with our pharmacokinetic analysis [77].

The current thioflavin-T assay and TEM analysis showed efficient inhibition of amyloid-β_1-40_ assembly. Preventing fibril formation and disentangling preformed fibrils by taxifolin has also been reported in previous investigations [60, 61]. The decrease in fluorescence intensity in the presented assay is likely to indicate reductions in amyloid-β oligomers and preformed fibril dissociation, as oligomers, in addition to amyloid-β fibrils, bind to thioflavin T [41]. The current assay indicated marked amyloid-β_1-40_ disassembly when 300 μM of taxifolin was added. Taxifolin is known to disassemble amyloid-β when auto-oxidated [61]. Oxidation of the catechol moiety of taxifolin results in a formation of o-quinone. Oxidized taxifolin is covalently bound to amyloid-β at Lys^16^ and Lys^28^ residues, which prevents amyloid-β assembly, as Lys^16^ and Lys^28^ residues are critical in β-sheet formation. Previous reports have shown that amyloid-β disassembly with taxifolin proceeds slowly [60, 61]. Thus, a lesser concentration of taxifolin may disassemble amyloid-β if incubated longer. Indeed, amyloid-β_1-42_ fibrils can be disentangled by addition of 50 μM taxifolin for more than 24 h [60]. We administered taxifolin to mice from the age of 1 month. Therefore, the long period of administration is likely to contribute to the disassembly of amyloid-β by relatively low dose of taxifolin administration compared to the results in vitro.

The pivotal role of amyloid-β oligomers in CAA has not been fully clarified, although the pathogenicity of soluble amyloid-β in CAA is widely known. Cerebrovascular function was impaired even before the appearance of vascular amyloid-β accumulation in Tg-SwDI mice, which were associated with soluble amyloid-β [22, 52]. Passive immunotherapy by ponezumab, intended to mobilize the interstitial fluid pool of amyloid-β, thus reducing soluble levels, resulted in amelioration of vascular amyloid-β accumulation and an improvement of CVR [5]. However, no linkage between CAA severity and amyloid-β oligomer expression was documented in a human autopsy study [67]. This may be attributable to limitations of biochemical analysis in human brain samples or bias resulting from the comorbidities of Alzheimer’s disease and CAA.

In the present investigation, cerebrovascular dysfunction in Tg-SwDI mice was drastically improved by taxifolin treatment, likely resulting from a reduction of toxic soluble amyloid-β species, including oligomers. Since soluble amyloid-β has been shown to increase oxidative stress and may result in cerebrovascular deficits [52], strong anti-oxidant activity of taxifolin may have also contributed to phenotypic recovery in Tg-SwDI mice. However, taxifolin did not change the level of cerebrovascular RAGE expression in Tg-SwDI mice. RAGE is the major amyloid-β influx molecule promoting amyloid-β transcytosis in the BBB [14, 15], suggesting taxifolin was unlikely to affect the AGEs-RAGE axis in Tg-SwDI mice. Taxifolin treatment did not affect CBF and CVR in WT mice, implying taxifolin possesses no additive effects on normal vessels. Thus, the drastic recovery of cognitive and cerebrovascular function shown in taxifolin-treated Tg-SwDI mice could stem mainly from inhibition of amyloid-β assembly.

The blood amyloid-β_1-40_ level was significantly elevated in taxifolin-treated Tg-SwDI mice. Taxifolin maintained cerebrovascular integrity in Tg-SwDI mice, which might facilitate vascular-mediated removal of amyloid-β from the brain [58]. Furthermore, disentangled amyloid-β may have been cleared more efficiently through transcytosis across BBB as well as the perivascular lymphatic drainage (PVD) system [3]. Interstitial fluid and solutes, including amyloid-β, are cleared from the grey matter through the PVD route, formed by two basement membranes in the walls of cerebral capillaries and arteries, into the cervical lymph nodes and systemic venous systems [9, 45, 46]. A congested PVD pathway could be one of the causes of CAA as amyloid-β distribution in CAA closely corresponds with the PVD route [73]. Soluble small molecules have a greater chance of being effectively cleared through the PVD route than large molecules [6]. Thus, increased levels of blood amyloid-β_1-40_ in taxifolin-treated Tg-SwDI mice may be attributable to promotion of amyloid-β clearance. We reported cilostazol, a selective type-3 phosphodiesterase inhibitor, ameliorated accumulation of amyloid-β through facilitation of the PVD route [42]. Synergistic effects may be expected when taxifolin and vasoactive drugs, such as cilostazol, are combined in CAA patients.

Some limitations of our study should be recognized. We analyzed only one strain of CAA model mice. Old-aged Tg2576 and other mice overexpressing APP also exhibited CAA [31], but the use of Tg-SwDI model has two distinctive advantages. Firstly, Tg-SwDI mice exhibit the vasculotropic amyloid-β_1–40_ dominant accumulations seen in the human CAA brain [63], although other transgenic models harboring mutant *APP* genes show abundant amyloid-β_1–42_ predominant in parenchymal plaques. Secondly, Tg-SwDI mice express the human transgenic APP at relatively low levels compared to other *APP* transgenic mice, similar to endogenous mouse APP [12]. Swedish/Dutch/Iowa mutant amyloid-β impaired its cerebrovascular-mediated clearance system resulting in robust amyloid-β accumulation within cerebral vessels [13]. Since decreased elimination, rather than increased production, of amyloid-β is likely to be a major cause of sporadic CAA, as well as Alzheimer’s disease [44], this model is useful for investigating mechanisms and therapeutic approaches in CAA [57, 58].

The protective effects of taxifolin against stroke were not confirmed in this study. Cerebral microinfarcts and microbleeds may cause cognitive impairments in patients with AD and CAA [11, 37]. Tg-SwDI mice exhibited relatively few cerebral infarcts and bleeds; however, chronic hypoperfusion has been shown to induce cerebral microinfarcts in Tg-SwDI mice [29, 42, 51]. Cerebral microinfarcts are preferentially distributed in arterial borderzone areas in hypoperfused Tg-SwDI mice, and are attributed to impaired CVR. The evaluation of CVR was adopted as a primary endpoint in a phase II clinical trial of ponezumab for probable CAA patients [54]. Since taxifolin fully recovered CVR, taxifolin may help prevent ischemic infarcts in CAA.

## Conclusions

Blocking amyloid-β assembly and oligomer formation provides a promising therapeutic target for CAA. Taxifolin maintained cerebrovascular integrity, and completely prevented cognitive decline in CAA model mice. Multifactorial data-driven analysis of information from the Alzheimer’s Disease Neuroimaging Initiative uncovered cerebrovascular dysregulation as an early pathological event in the development of Alzheimer’s disease [34]. Since the mechanisms behind pathogenesis in Alzheimer’s disease and CAA are closely overlapped [59], therapeutic intervention for CAA may also provide a novel disease-modifying therapy for Alzheimer’s disease. A prospective clinical trial is urgently required to determine the effects of taxifolin on CAA and Alzheimer’s disease.

## Abbreviations

AGEs: Advanced glycation end products
BBB: Blood-brain barrier
CAA: Cerebral amyloid angiopathy
CBF: Cerebral blood flow
Cmax: Maximum concentration
CVR: Cerebrovascular reactivity
ELISA: Enzyme-linked immunosorbent assay
FITC: Fluorescein isothiocyanate
PVD: Perivascular lymphatic drainage
RAGE: Receptor for advanced glycation end-products
TBS: Tris buffered saline
TEM: Transmission electron microscopy
WT: Wild type
